# Exploration of nature-based biomimetic approach in landscape architectural design: parametric study of candelabra model design

**DOI:** 10.1186/s42492-020-00060-y

**Published:** 2020-11-04

**Authors:** Biljana S. Jović, Anđela D. Mitić

**Affiliations:** Department of Landscape Architecture, Faculty of Forestry, University of Belgrade, Belgrade, 11000 Serbia

**Keywords:** Biomimetic approach, Landscape architectural design, Parametric study, Blender software, Three dimensional model of candelabra

## Abstract

This research provides an exploration of a biomimetic approach in the process of designing a candelabra model using linear shaped leaves of a Bell flower. The design process described in this research contains two steps: biological and geometrical. In the first biological step, a proper model for the creation of an urban element was found from nature in a Bell flower (*Campanula persicifolia* L.). The upper leaves of the selected plant, which are small with a linear spear and sharpening at the top, were chosen for the modeling process. The second step included applying two geometrical methods, i.e., Voronoi diagrams and Delaunay triangulation. A geometrical leaf form of the selected plant species and the modeling process were obtained using aparametric modeling software, Blender. Using different Blender plug-ins and modifiers, Delaunay triangulation and Voronoi diagram were implemented by marking the starting points on the leaf form in the image data source, adjusting the Delaunay triangulation parameters, and creating Voronoi diagrams in which the Voronoi points were located at the shortest distance from the edges of the Voronoi polygon. Consequently, a three dimensional model of a candelabra was developed through this study.

## Introduction

Biomimetic approach presents a design tool that interprets the natural forms and transfers them into the original design solutions [1]. The application of a biomimetic design method is a relatively new approach in architecture, landscape architecture, and engineering and has been used in various design solutions in different fields. This novel and original design solution can represent new values in our environment and have a positive impact on mental health.

The imitation of various organic forms, resulting in new and original elements and values of our environment, represents the basis of the biomimetic methodology. The properties of living structures and forms that exist or act in nature are used in the creation of various designs according to biological principles [2]. By applying different geometric principles and biological knowledge to the process of modeling and copying models from nature, it is possible to produce various structures [3].

According to Pedersen, there are two categories in which biomimetic approaches as a design process typically belong [4]. The first category, called design looking to biology, means defining a human need or design problem and looking for an answer in a system existing in nature. The second category is defined as a biology-influencing design, which means identifying a particular characteristic or function in an organism or ecosystem and translating it into a human design [4]. In this research, the influence of biology on the design concept was considered.

The exploration of this nature-based approach (as first applied by Da Vinci [1]) and its application to a parametric design starts by defining the two main aims of this research. The first aim is to explore the utilization of a biomimetic approach in the modeling process of single element often used in landscape-architectural projects. The second aim is to investigate the parametric modelling based on the image source of the model chosen in nature and geometrical methods such as the Voronoi diagram and Delaunay triangulation in the popular open-source three dimensional (3D) computer graphics software, Blender.

The research starts by selecting an appropriate plant species as a model from nature, which satisfies two main requirements: (1) using a simple and linear leaf form and (2) applying a widely known species. The purpose was to design an open space element from a model in nature that can be relatable for people. The inspiration chosen for this research was the Bell flower (*Campanula persicifolia* L.).

A theoretical review of the biomimetic and biomimetical approaches through parametric modeling of different designs is provided in Theoretical review section. Methods section presents a parametric study on the design of a 3D candelabra model based on a model found in nature using Blender. Subsequently, the results of this study are presented in Results and discussion section. Finally, in the last section, future directions of the biomimetic approach using parametric modeling as a tool in the design of new and healthy environments are provided.

## Theoretical review

The term ‘biomimetic’ is derived from two Ancient Greek words: bios (βίος), which means life, and mīmēsis (μίμησις) or mīmeisthai (μιμεῖσθαι), which means imitation or to imitate [5]. The term represents the imitation of various models or systems of nature resulting in new and original elements of our environment. Another term with a similar meaning is biomimicry, which was introduced in 1950 by Otto Herbert Schmitt (1913–1998), an American inventor, engineer, and biophysicist. Schmitt wanted a term to describe the transfer of ideas and analogies from the domain of biology to technology [6].

The nine principles of biomimicry (biomimetic) defined by Janine M Benyus primarily represent ways in which nature functions, i.e., nature runs on sunlight; nature uses only the energy it needs; nature fits form to function; nature recycles everything; nature rewards cooperation; nature banks on diversity; nature demands local expertise; nature curbs excesses from within; and nature taps the power of limits [7]. These principles of nature were complemented and expanded by the Biomimicry Institute, which postulated six major biomimicry principles and their constituting 23 principles [8]:

1. Resource (material and energy) efficient;
2. Evolved to survive;
3. Adapted to changing conditions;
4. Integrated development with growth;
5. Locally attuned and responsive;
6. Uses eco-friendly chemistry.

Biomimetic is a field that has potential to provide various solutions to deal with major global challenges. The quest for sustainable solutions to human challenges has pushed scientists, engineers, architects, designers, and innovators to learn from the elements of nature [8].

A biomimetic approach can make a difference in all aspects of design. A biomimetic approach can offer solutions to many of the 15 global challenges defined by the Millennium Project, including sustainable development, water supply, information technology, health, energy, and science and technology. The researcher Ingo Rechenberg envisioned that, by 2099, a biomimetic world will be implemented through material science, production technologies, energy systems, mobility, sensing technologies, recycling, robotics, sports, computer science, and even politics [9].

**Fig. 1:**
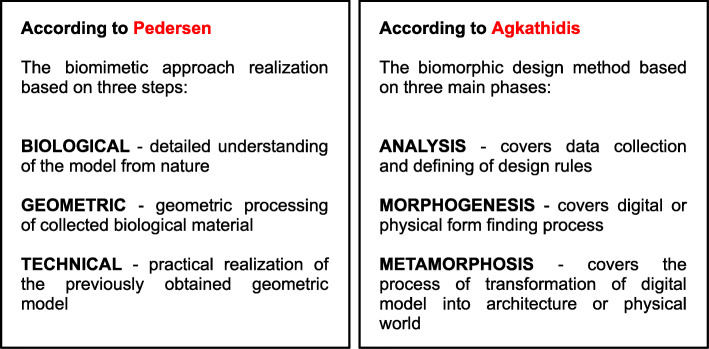
Scheme of two biomimetic approaches

A practical realization of the biomimetic approach (biology influencing design concepts) takes place in three steps: biological, geometric, and technical [10] (Fig. 1). For a detailed understanding of a model, the biological step includes the collection and use of knowledge from different areas including botany and zoology. All such knowledge is necessary to further establish a relationship with the geometrical and technical steps of the biomimetic approach. Further, a detailed analysis is used to find the appropriate model from nature, followed by a geometric step. A geometric step includes the geometric processing of the collected biological materials. It approaches the modeling process by providing appropriate geometric methods that can be used to define the form of a nature-based model. The technical step aims to provide a practical realization of the previously obtained geometric model.

Another example of the biomimetic approach is given by Agkathidis in a study on the implementation of a biomorphic design (Fig. 1). The term ‘biomorphism’ was derived from Goethe and is composed of two Greek words, βίος (meaning life or living) and μορφή (meaning form) [11]. Biomorphism is related to nature-inspired forms and patterns, and shapes in art, architecture, and design. The design method created by Agkathidis was based on three main phases: analysis, morphogenesis, and metamorphosis [12]. The first phase, analysis, covers data collection and defining of design rules, similar to the first step—biological phase—of Pedersen’s biomimetic approach. The second phase, morphogenesis, covers a digital or physical form finding process similar to the second step, i.e., the geometric phase, of the biomimetic approach applied in this research. The results of this phase can be represented as prototypes or generative models. The final phase, metamorphosis, covers the process of transformation of digital models into a physical world. This phase is similar to the final step, i.e., the technical phase, of the biomimetic method.

Parametric tools are algorithmically based and offer computational control over the design geometry. Parametric modeling is extremely useful for design exploration under different design settings [13].

Several researchers agree that the term ‘parametric’ originates from mathematics. Some researchers consider that the application of the parametric approach started in 1978 when a system for combining two parameters (dimensions and tolerances) to design mechanical components was created. However, other researchers believe that the parametric approach was introduced earlier in the 1940’s [14].

The parametric approach in an architectural design can be implemented in two different types of parametric software, i.e., conceptual and constructive parametric designs [15].

In the first design, the parameters of a particular design, excluding the shape, are declared. This can be achieved by assigning different values to the parameters, and different objects can be created. Software used for this type of parametric design includes Maya (designed primarily for the film industry) and Rhinoceros [15].

A constructive parametric design refers less to parameters and more to data embedded within a predetermined 3D object. The software through which this group of designs are created includes CAD software such as Autodesk Revit, Soft Plan, Nemetschek, ArchiCAD, and Chief Architect, all of which are BIM-based, for which the designer can use pre-defined components such as doors and stairs [15].

There are numerous examples of conceptual parametric designs. A similar study on the design of an urban bench was generated using a 3D model in Rhinoceros, and the structural skin was applied through the popular Grasshopper plug-in [16]. Another study using Rhino software and the Grasshopper plug-in was conducted to model stadium facades based on the geometric parameters of the eye. This study explored the interactions between building facades and the environmental conditions in an energy-efficient building design [17].

There are numerous software programs that can be used for parametric modeling. For this research, we chose an open-source software, which is continuously upgraded and provides new modeling experiences. Blender software is an open-source 3D computer graphics modeling and animation program maintained by the Blender Foundation. It is a comprehensive software with limitless possibilities in terms of modeling and animation [18].

**Fig. 2:**
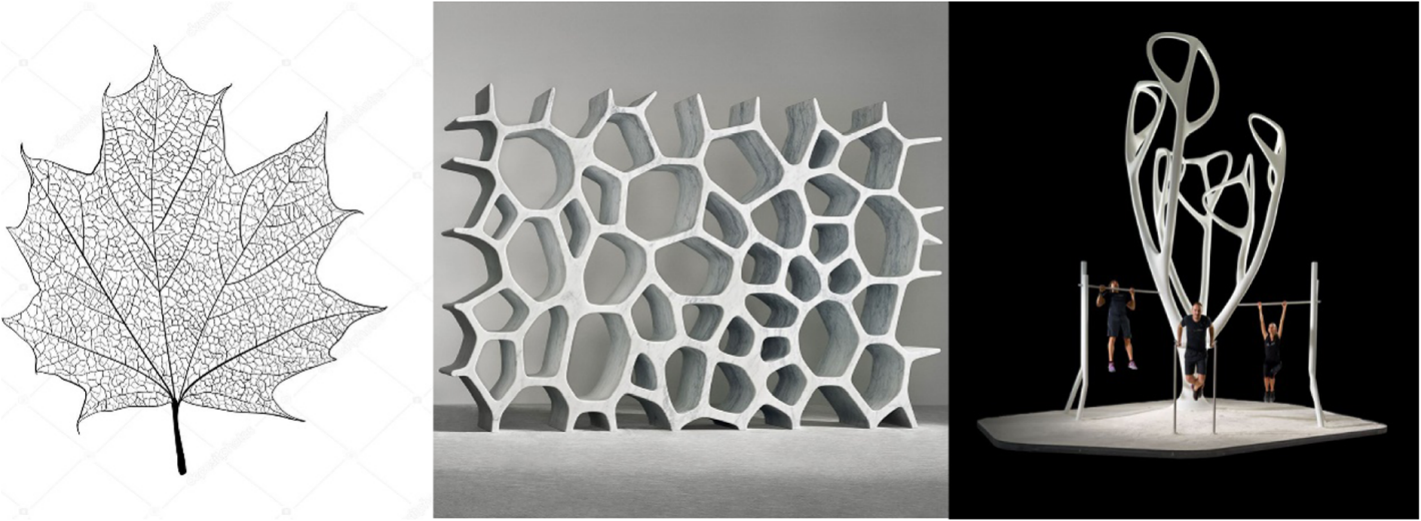
Three steps of biomimetic approach for the example “My Equilibria”, an urban furniture design (source: http://www.myequilibria.com/myequilibria/ and Anđela D. Mitić)

**Fig. 3:**
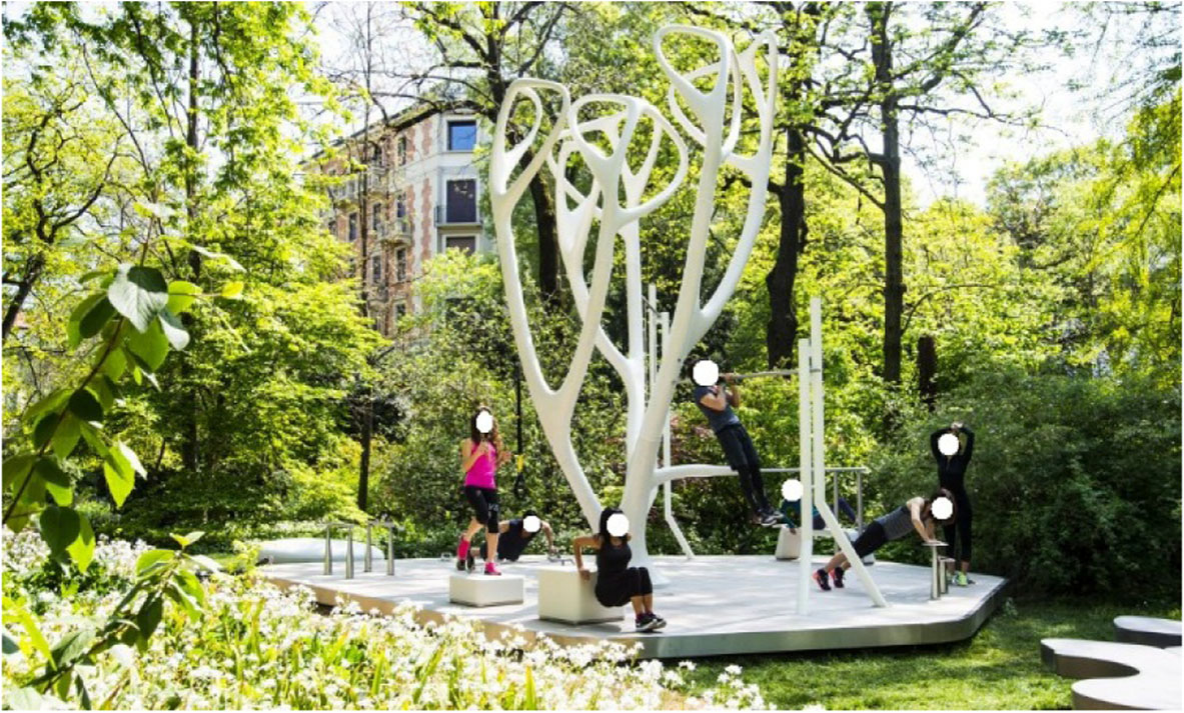
Element of urban space, “My Equilibria”, designed by Vito Di Bari and Alfredo Tasca (source: http://www.myequilibria.com/myequilibria/)

Example applications of a biomimetic approach in parametric modeling, as defined by Pedersen, can be seen in the design of urban furniture by the authors Vito Di Bari and Alfredo Tasca. The urban furniture element, My Equilibria, is a part of an outdoor fitness installation concept that redefines the workout experience and breaks the boundaries between art, technical equipment, and the local community [19]. For the biological step, inspired by nature, a nervure of leaves is applied. As a geometrical method, a Voronoi diagram was used for obtaining the proper geometric form for the modeling process (Fig. 2). Further, the model was technically solved, and it currently represents an urban furniture used for outdoor fitness workouts (Fig. 3). There are several examples of modeling different elements in architectural and landscape-architectural projects [10, 16, 17, 20, 21], as well as in other professions, including clothing production [22]. Biomimetics can help improve the ecological footprint in the clothing sector by inspiring innovative designs through the clever use of materials and different structures in the functional aspect of clothing [22].

## Methods

**Fig. 4:**
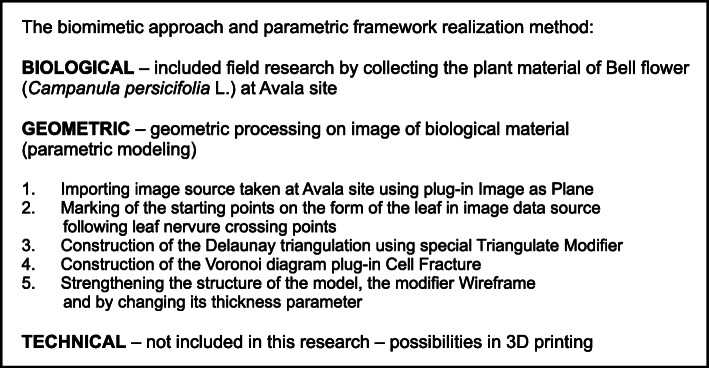
Framework of the method used in this research

**Fig. 5:**
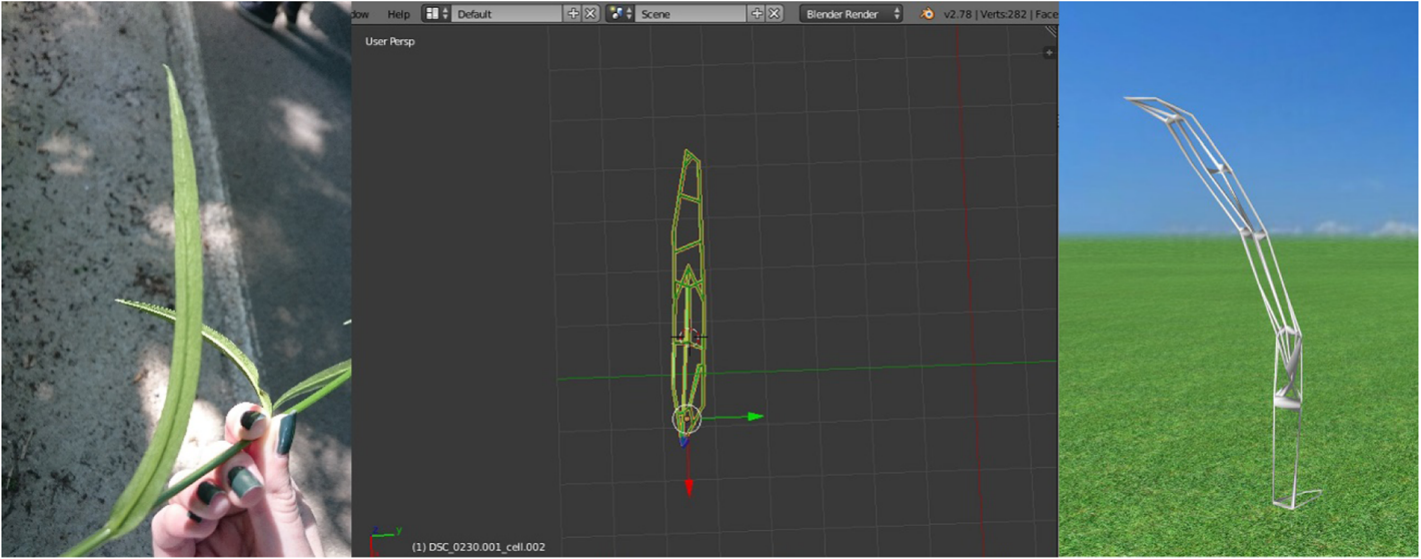
Design of a 3D candelabra model using a biomimetic approach

The method used in this research includes field research applied in the collection of Bell flowers and the use of Delaunay triangulation and the Voronoi diagram as geometric methods applying Blender software, as well as the modeling of a leaf selected as a corresponding model from nature. All of these methods can be presented through a three-step biomimetic approach for the design of common urban elements found in our environment (Figs. 4 and 5).

The biological stage covers the discovery of an appropriate model from nature. The chosen model should satisfy two main requirements: (1) a simple and linear form of a leaf and (2) a member of a widely known species. The first requirement was important because the form of the candelabra needed to be simple with long vertical lines for fitting its form to function [i.e., resource (material and energy) efficient based on the biomimetic principle]. For the second requirement, we needed a plant species that is widely known so that it is relatable to a 3D model. In addition, for the second requirement, it was important that the plant be found at the Avala site, a mountain near Belgrade, Serbia, where people often go on picnics or for jogging or hiking. This was significant because the purpose of this research was to design an open-space element from the natural environment near Belgrade, Serbia, through which people can relate.

The Bell flower (*Campanula persicifolia* L.), an herbaceous perennial plant usually reaching heights of up to 1 m, was chosen, which satisfied all requirements of this research.

**Fig. 6:**
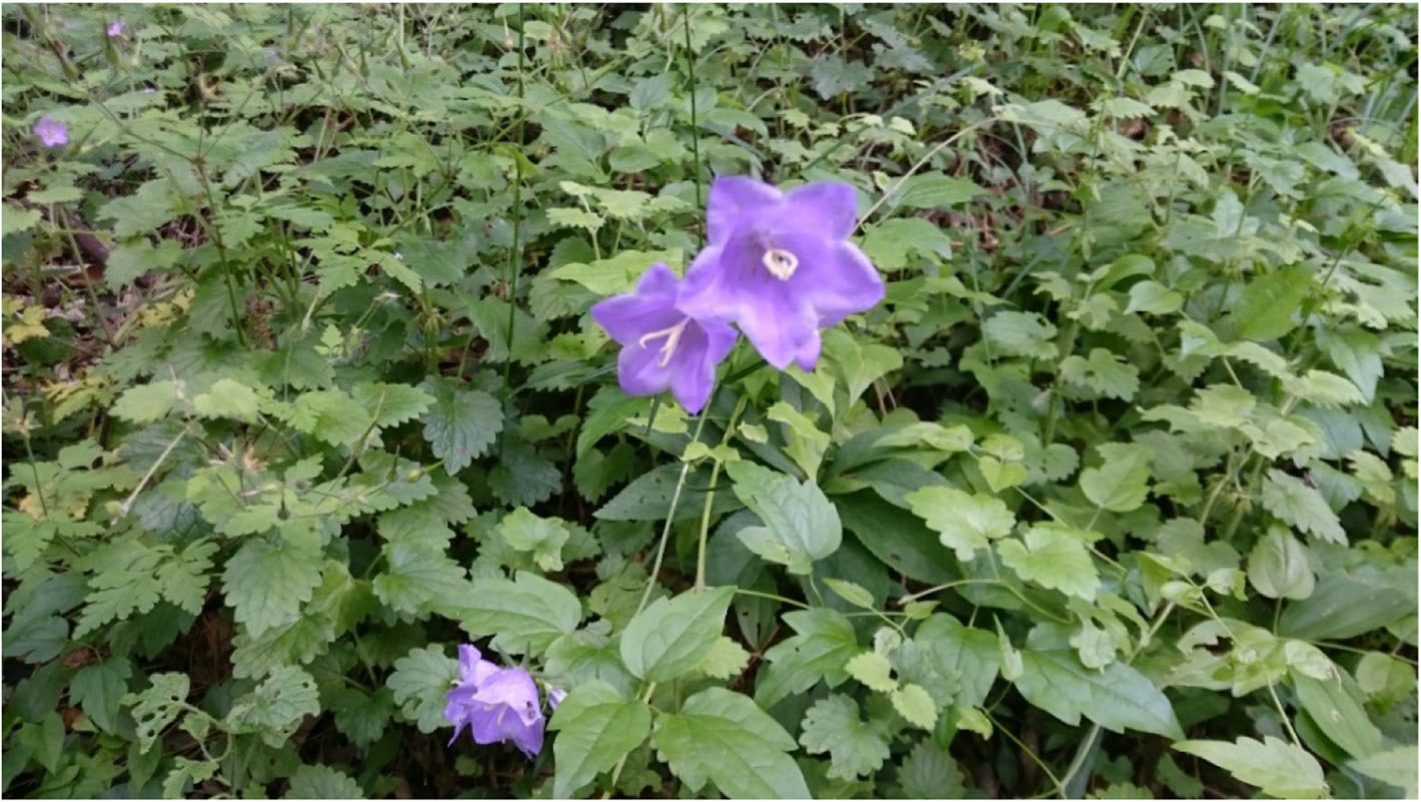
Bell flower (*Campanula persicifolia* L.) at Avala site, Belgrade, Serbia

**Fig. 7:**
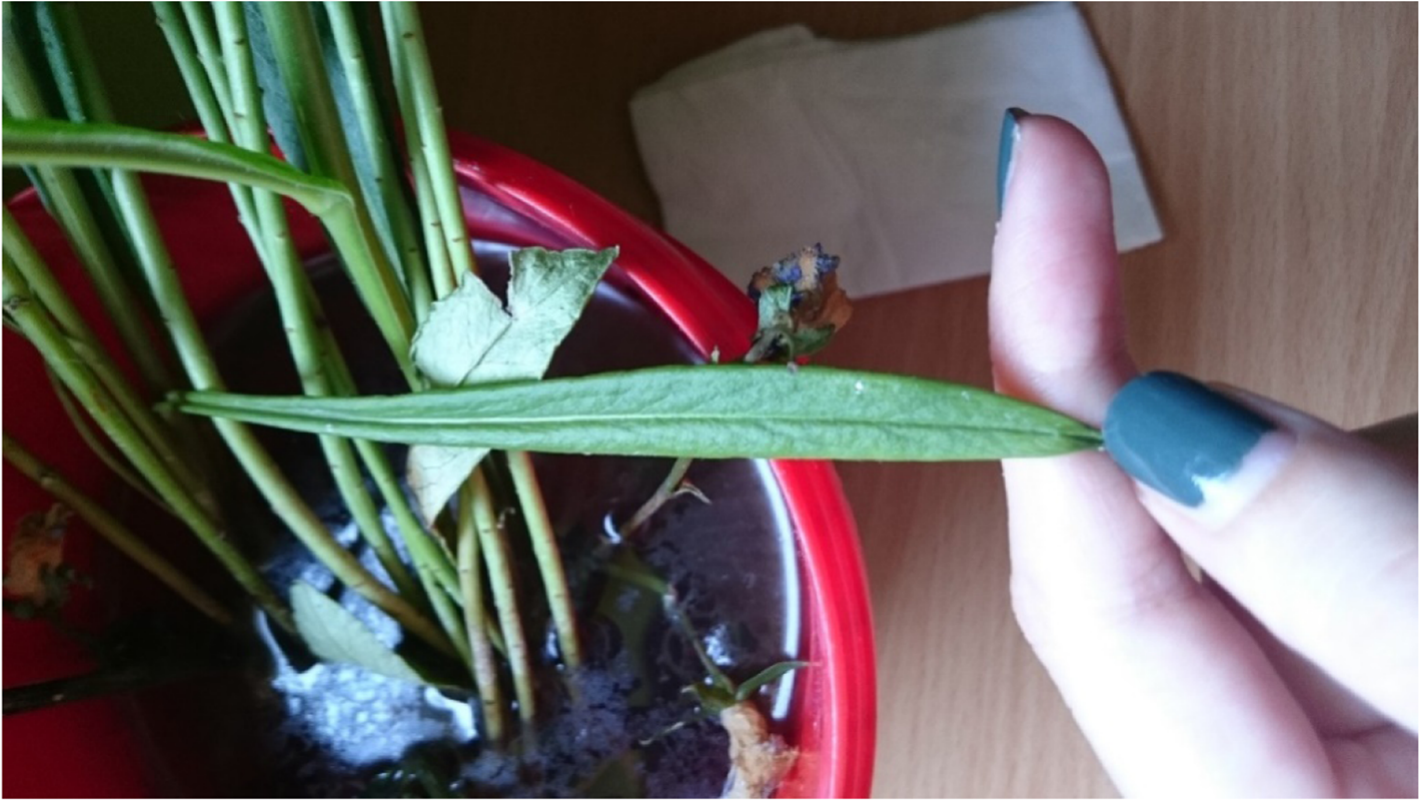
Leaf of selected plant species: Bell flower (*Campanula persicifolia* L.)

It was initially described in 1753 by Linnaeus, followed by Professor Josif Pančić in 1874 in Serbia. It has a Mediterranean origin and can be found in Querco-Fagetea and Quercetalia pubescentis forest types. Owing to its flowering period, which is in April and May, field research was conducted at the Avala site near Belgrade on May 27, 2017. The lower leaves of a bell flower are simple, elongated, and narrowed, whereas the upper leaves are smaller, with a linear and sharp spear shape at the top (Figs. 6 and 7) [23].

To apply a geometric step and modeling of the corresponding form, geometric methods of Delaunay triangulation and the Voronoi diagram through Blender were used. These geometric methods were selected because of their common use in parametric modelling and nature-based patterns, particularly the Voronoi diagram. As a basic parametric software, Blenderhas different tools such as modifiers and parameters within the software package that allow us to manipulate and modify the model and its characteristics until we obtain the desired result [24].

**Fig. 8:**
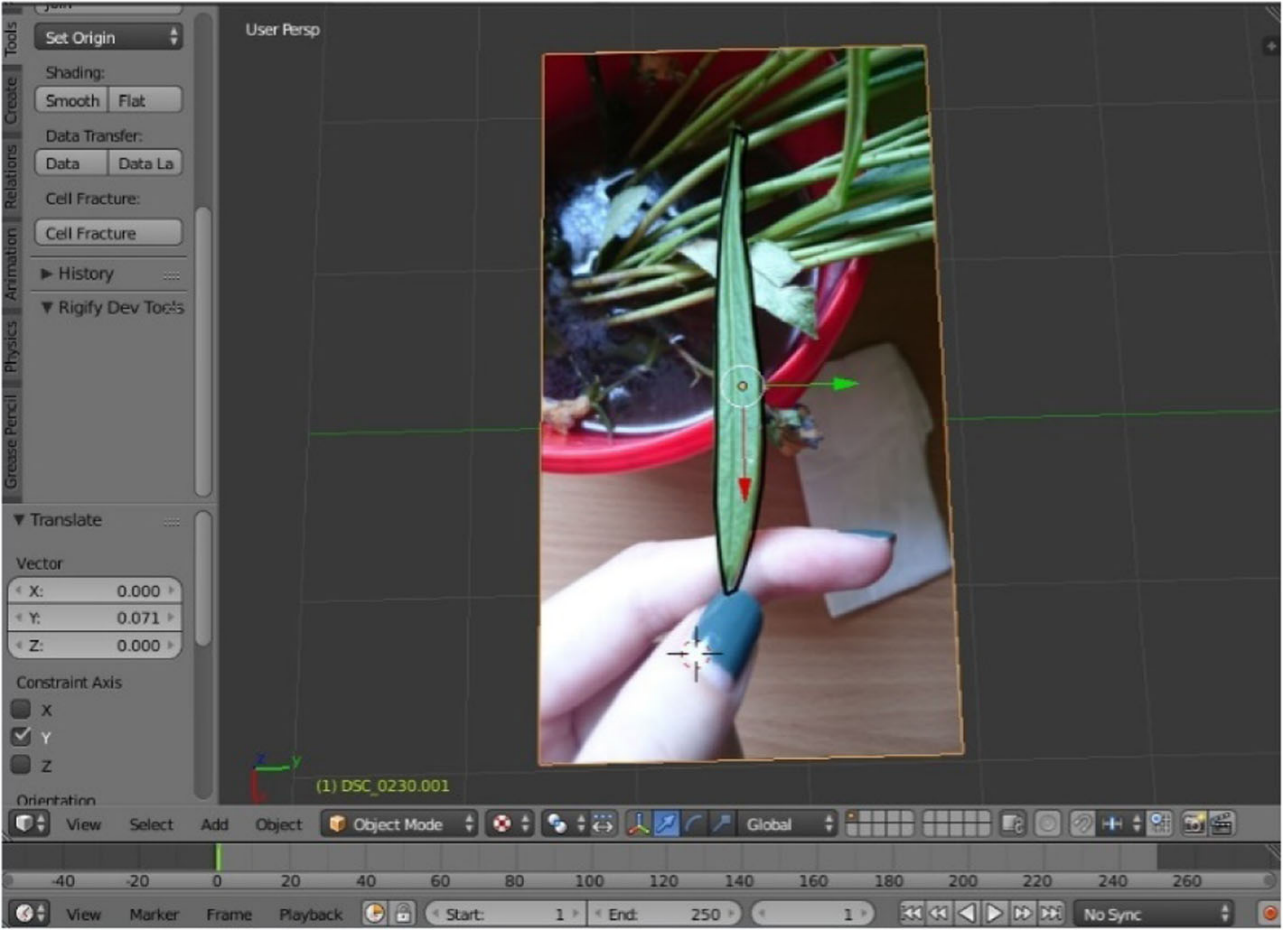
Importing selected image source

**Fig. 9:**
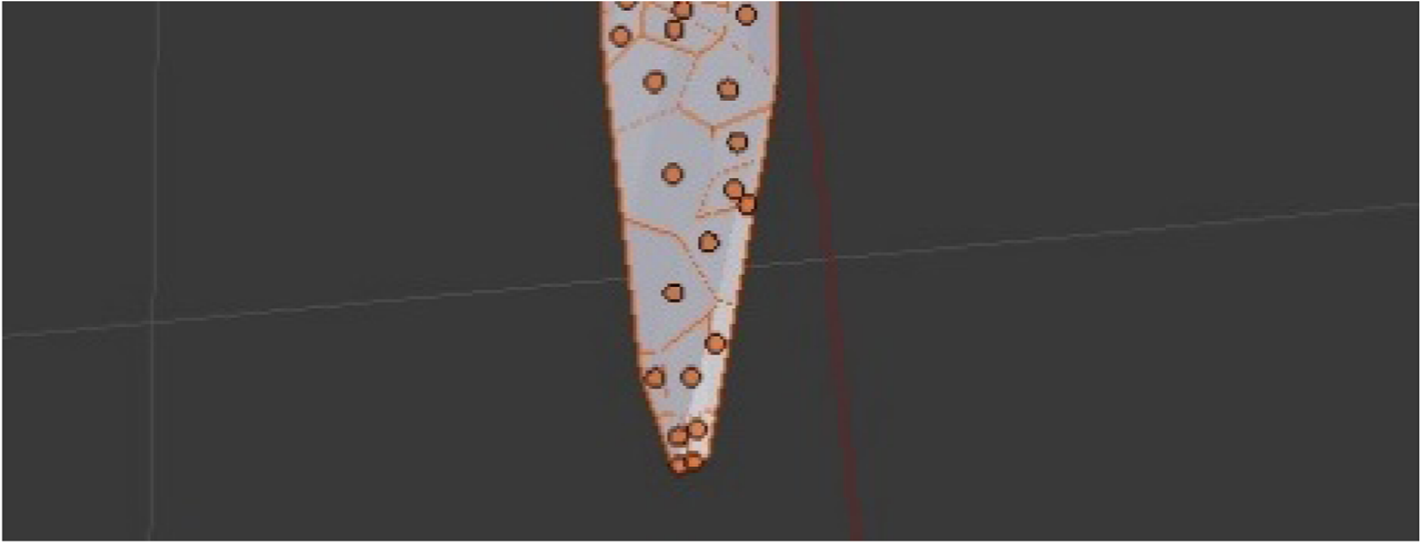
Results of applying Triangulate Modifier

**Fig. 10:**
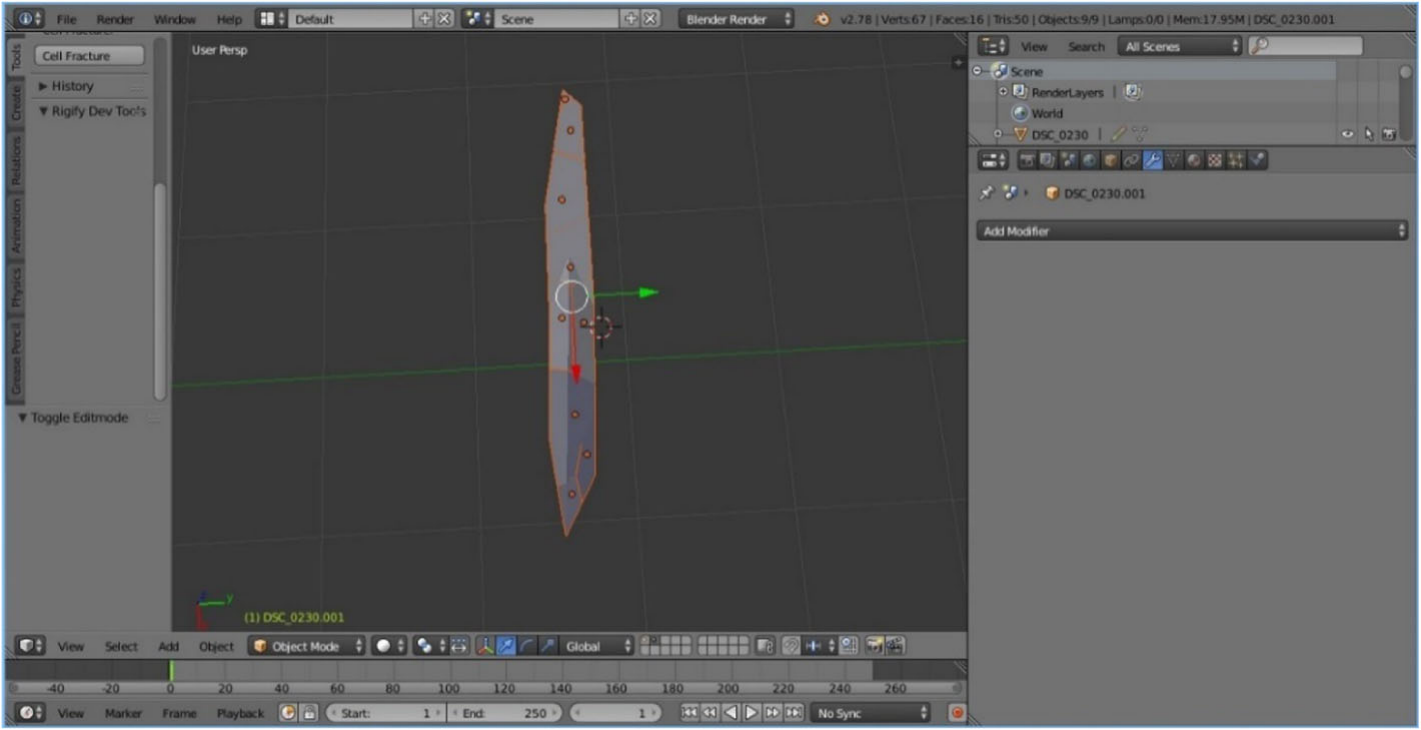
Construction of Voronoi diagram

The modeling process starts with importing a selected image of the selected species upon which the mentioned geometric methods have been applied, including the chosen plug-ins and their parameters. The first step was importing the selected image source captured on site using the Image as Plane plug-in (Fig. 8). The second step was the construction of Delaunay triangulation, which covers the process of joining the closest neighbor points in the longitudinal direction and determining half of the line segment, thereby constructing the normal direction at the point of the spin and determining the intersection point. This was applied using a Triangulate Modifier (Fig. 9), which is one of the useful modifiers in Blender, and it was built according to the Delaunay triangulation geometric method with a few upgrades. The creation of a Voronoi Diagram consists of marking the starting points in which Voronoi points are located at the shortest distance from the edges of the Voronoi polygon [10] (Fig. 10). This was achieved using a cell fracture plug-in, which can easily transform future models.

**Fig. 11:**
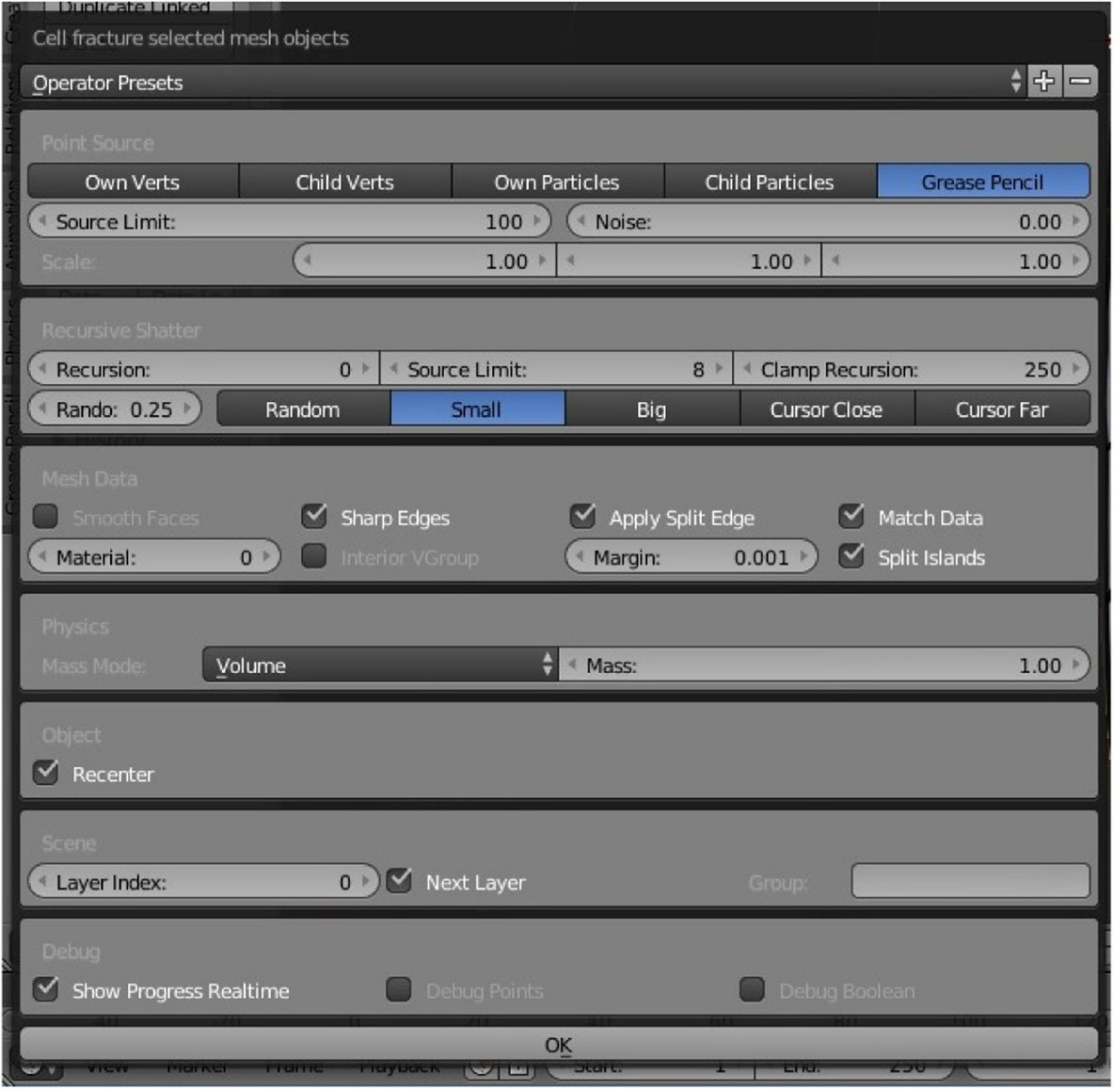
Implementation of Cell Fracture plug-in in Blender software

A 3D model was created through Blender, by employing plug-ins such as Image as Plane and Cell Fracture (Fig. 11) and generating a geometric pattern of the nervure of a leaf (Bell flower).

**Fig. 12:**
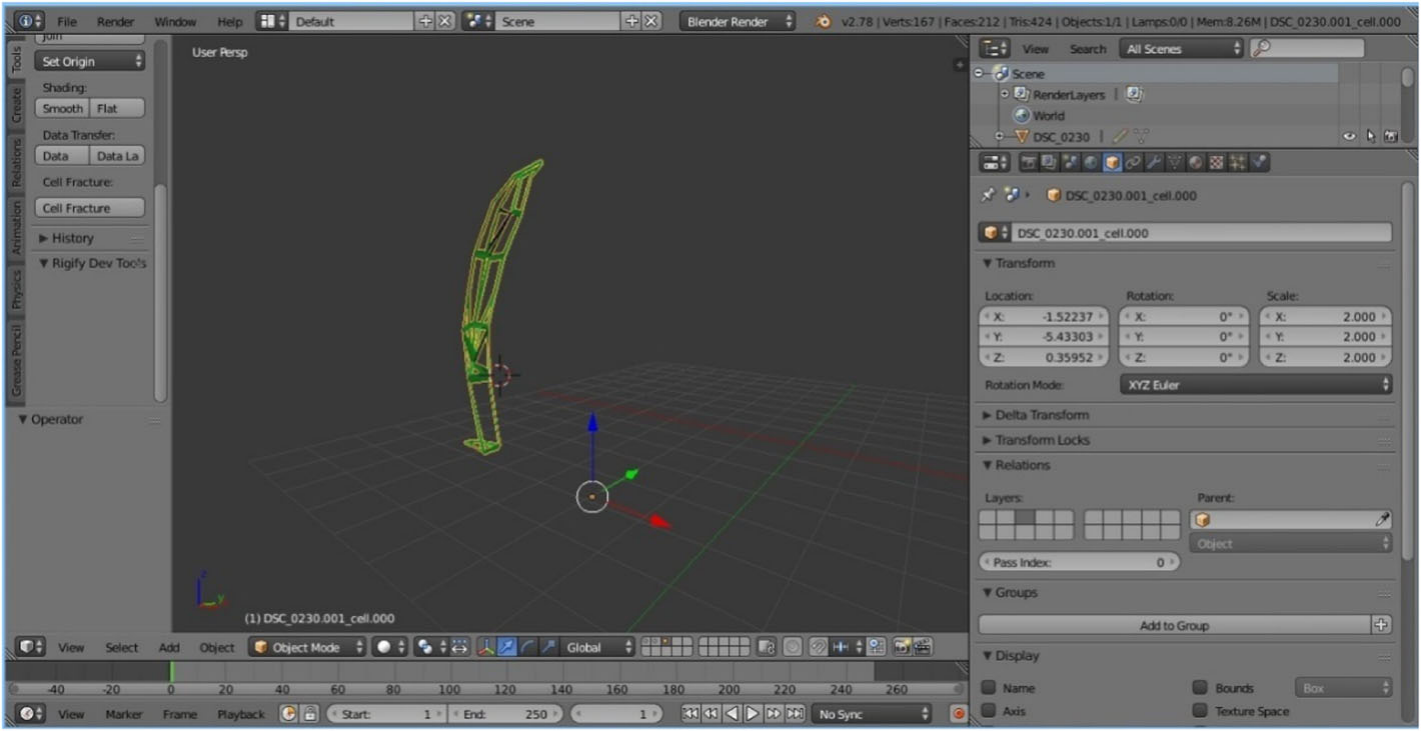
The model of a candelabra obtained in Blender

During the process of strengthening the structure of the model, the Wireframe modifier was used as the last step of the modeling process by changing its thickness parameter. Subsequently, the obtained model was a frequently-used urban element for lightening, called candelabra (Fig. 12).

## Results and discussion

**Fig. 13:**
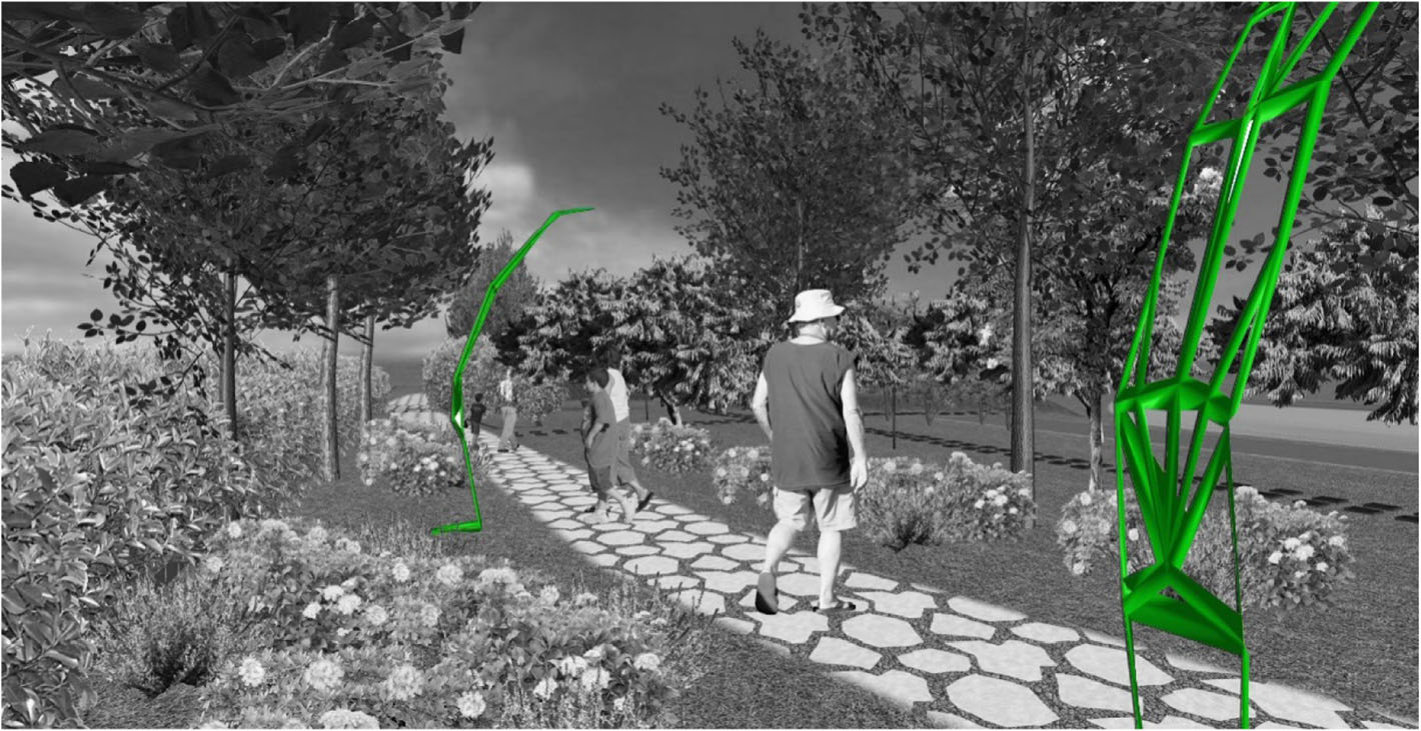
Conceptual design of candelabra

Based on the leaf of the Bell flower and by using the modifiers and their parameters in the Blender during the process of applying the biomimetic approach, this research resulted in a conceptual design of an urban element often used in landscape architectural design (Fig. 13). The construction of this urban design model was presented as a 3D candelabra model.

This research included the implementation of a two-step biomimetic approach. The first biological step was to identify a plant species satisfying all the requirements. The modeling process started with importing the selected image of a plant. In Blender, there is a special plug-in for importing images, which can be limiting due to this option being provided in the main menu in other software. To apply the selected geometric methods, all modifiers and plug-ins used by Blender needed to be researched because there are several ways through which the results of this study could be obtained. However, in this study, only one parametric (geometry) method was represented and few modifiers and plug-ins of Blender were used.

For the third step, i.e., the technical step, 3D printing technologies can be used in future research. Blender can also be used for converting any design into a printable 3D file. The process of preparing a 3D model for printing includes adjusting the scales and units, activating the mesh plug-in of the 3D Print Toolbox, and polygonal modeling corrections of the model (checking the volumes of the thickness parameter and minimizing the number of polygons). In addition, Mesh Analysis is a verification tool used for checking a 3D model. The last step is exporting a monochrome 3D print in an .stl file format (stereolitography) or a multicolor 3D print as an .obj or .dae file format, using the file–export path [24].

**Fig. 14:**
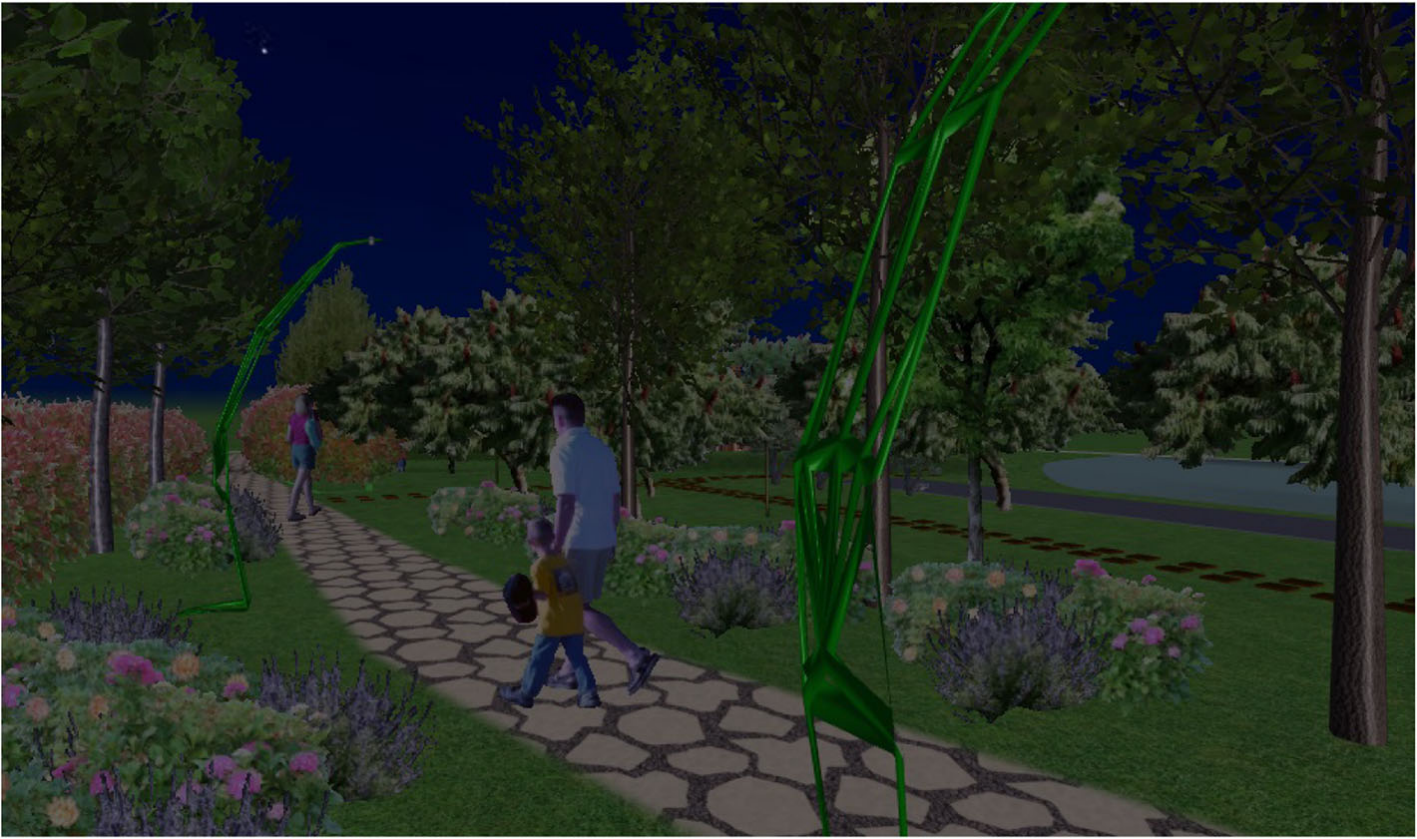
Candelabra model in nighttime environment

This urban design model can be presented as an original, innovative, and attractive element of every open urban space (Fig. 14). As a model obtained using a biomimetic approach, it can also induce a new aesthetical experience in its users within an urban space. In addition, it can strengthen the connection with nature by introducing and returning some of nature’s forms and patterns into an urban environment.

The biophilia hypothesis implies that humans have an affection for all natural elements, such as plants and other living things. In addition, this hypothesis considers that the implementation of natural elements can be beneficial for human health. Many studies have documented these hypothetical experiences. It is considered that nature and all of its elements can have psychological benefits on human health, such as reducing stress and improving focus, with a positive effect on mental restoration and other factors. There have been numerous studies indicating that simply viewing natural landscapes can provide health benefits, particularly stress reduction [25]. Velarde et al. [26] reported that natural landscapes have a positive health effect on people, whereas urban landscapes largely have a negative effect. Numerous studies have confirmed the possibilities of a positive visual experience and the effects of an aesthetic value from a natural environment [27, 28]. Based on an assessment of the impact of three parks characterized by their level of biophilic quality, it was reported that the perceived level of restoration is associated with the sense of connection to nature [28]. Biomimetic principles in a landscape architectural design can improve the physical environment of society.

Ecosystem biomimicry and its principles can have a valuable role in adaptation to climate change, particularly in constructed environments [29]. In addition, biomimetic aspects can influence economic development [30]. A similar perspective was framed by the idea of biomimetic capitalism [31].

## Conclusions

This research was aimed at presenting the integration of a biomimetic approach into the landscape architectural design process demonstrated by a minimalistic 3D model design of a candelabra. The biomimetic approach combined with parametric modeling tools used in the popular computer software Blender represents a method for the design of a 3D model inspired from nature. Owing to the Blender community, this software provides several new tools each month. Today, Blender has the same node environment for coding as Grasshopper. For further studies, exploring this new environment in Blender and comparing the modeling process with Grasshopper could be beneficial. In addition, the possibilities of using 3D printing as a third step of this research method could be explored.

By applying this innovative biomimetic approach in the design process, landscape architectural design solutions can continue to push and change the boundaries of form and construction in all their aspects. There are many positive effects when including the biomimetic approach as a new methodology into landscape architectural designs. These original design models of open spaces can be a novel direction toward a nature-inspired urban design, thereby creating new urban environments that strengthen the connection between people and nature. Today, we have numerous types of different ‘green’ concepts used to create harmonious urban spaces by introducing various natural elements affecting positively on the residents and the community in general.

## Data Availability

The datasets used and/or analyzed during the current study are available from the corresponding author on reasonable request.

## Abbreviation

3D: Three dimensional
